# Recognizing the Effects of Language Mode on the Cognitive Advantages of Bilingualism

**DOI:** 10.3389/fpsyg.2018.00366

**Published:** 2018-03-20

**Authors:** Ziying Yu, John W. Schwieter

**Affiliations:** ^1^Department of English Language and Literature, Fudan University, Shanghai, China; ^2^Department of Linguistics, University of California, Santa Barbara, Santa Barbara, CA, United States; ^3^Language Acquisition, Multilingualism, and Cognition Laboratory, Wilfrid Laurier University, Waterloo, ON, Canada; ^4^Bilingualism, Translation, and Cognition Laboratory, University of California, Santa Barbara, Santa Barbara, CA, United States

**Keywords:** language mode, language activation, cognitive benefits of bilingualism, language control, multilingualism

## Abstract

For bilinguals, it is argued that a cognitive advantage can be linked to the constant management and need for conflict resolution that occurs when the two languages are co-activated (Bialystok, 2015). Language mode (Grosjean, 1998, 2001) is a significant variable that defines and shapes the language experiences of bilinguals and consequently, the cognitive advantages of bilingualism. Previous work, however, has not sufficiently tested the effects of language mode on the bilingual experience. In this brief conceptual analysis, we discuss the significance of language mode in bilingual work on speech perception, production, and reading. We offer possible explanations for conflicting findings and ways in which future work should control for its modulating effects.

## Introduction

The claim that the knowledge and use of multiple languages gives rise to cognitive benefits is a hotly debated area of research in psycholinguistics and bilingualism (see Barac et al., 2014; Blom et al., 2017 for recent reviews). At any given point in time, and based on numerous psychosocial, situational, and linguistic factors, a bilingual must decide which language to use and how much of the other irrelevant language must be controlled or suppressed (Green, 1998). It is this constant management and monitoring of more than one language system that may be most responsible for the reported advantages in general executive functions (Bialystok et al., 2012; but see Hilchey and Klein, 2011; Paap et al., 2015 for alternate views).

Although a bilingual’s two languages are constantly in a state of co-activation, Green and Abutalebi’s (2013) Adaptive Control Hypothesis argues that the relative degree^1^ of such activation for each language is dynamically adaptive. This hypothesis builds on the fact that bilinguals vary regarding language use in several contexts (Green, 2011; Prior and Gollan, 2011) and links this variation with underlying cognitive and neural control mechanisms. Green and Abutalebi (2013) argue that the control mechanisms adapt in response to bilingual experiences and to the recurrent demands placed on them in interactional situations. It might be the case that bilinguals outperform their monolingual counterparts in some cognitive tasks because this advantage may be a representation of their superior ability to be adaptive to situational (e.g., experimental) needs. If this is the case, various experiences with language mode may confound the implications for a bilingual advantage. In order to study the nature of these effects more accurately and directly, researchers must take into account speakers’ language experiences thoroughly (Luk and Bialystok, 2013; Schwieter and Ferreira, 2016). These experiences are modulated by the limited capacity and goal-directed selectivity of the human executive functions.

Grosjean (1998, 2001) proposed and developed a notion of *language mode* which refers to the state of activation of the bilingual’s languages and language processing mechanisms at a given point in time. In other words, language mode concerns the degree of activation of the two languages in a bilingual’s mind. According to Grosjean, due to the influence of the environment, bilinguals continuously and naturally find themselves on a situational continuum of language activation, ranging from a monolingual to a bilingual mode. Three hypothetical positions can be visualized in this framework. When the bilingual is said to be in or close to being in a *monolingual mode*, a base language (i.e., the primary language being processed or produced at the time, not necessarily L1 for a bilingual) is the most active in terms of environmental activation (since the base language can also be L2 for an unbalanced bilingual), while the other non-target language is much less activated (but never totally deactivated). When the bilingual moves on the continuum and stops at an *intermediate mode*, the non-target language is more active than in the monolingual mode, whereas the base language remains the most activated language. When the bilingual is in a *bilingual mode*, in which the two languages are utilized from time to time in the form of code-switching or borrowings, the non-target language is highly active (but not as active as the base language). At all three positions, the base language remains fully active, as it is the main language that governs language perception and production.

Language activation is modulated by several variables including participants’ characteristics. While language mode focuses on environmental characteristics, participants’ characteristics including language proficiency and language dominance may change the activation level of languages. Dunn and Fox Tree (2014, p. 611) argued that “although there was no interaction between language mode and bilingual dominance, language mode can be made clearer when bilingual proficiency is controlled.” Consequently, we discuss participants’ characteristics in this paper in cases where researchers are careful to minimize their confounding influence.

Given that language mode plays an important role in language activation, it likely should be considered a modulating factor in the bilingual advantage debate. However, these possible effects have been unintentionally ignored, oftentimes by employing experimental designs that place and maintain participants in an intermediate mode. This misrepresents the true bilingual experience which consists of diverse interactions with and placements on the language mode spectrum and consequently uncovers findings that may be ambiguous or conflicting. Below, we discuss the important role of language mode in research on bilingual language activation, including speech perception, speech production, and reading. We offer ways in which studies investigating the cognitive advantages of bilingualism can consider the role of language mode.

## Speech Perception

In the area of bilingual speech perception, bilingual participants from some studies (e.g., Spivey and Marian, 1999; Colomé, 2001) may have been closer to an intermediate mode on the language mode continuum. Although the researchers examined one base language throughout the experiment, a number of confounding factors may have activated the participants’ other languages, consequently moving them away from the monolingual endpoint. Although it is difficult and perhaps impossible to place a bilingual in a complete monolingual mode throughout a task, a few experiments have attempted to control for language mode. In a lexical decision task, Soares and Grosjean (1984) compared Portuguese-English bilinguals’ reaction times (RTs) to words and non-words in English monolingual mode, Portuguese monolingual mode, and code-switching bilingual mode. Their results demonstrated that bilinguals were slower to access code-switched words in the bilingual mode than they were for words in the monolingual modes.

Similar findings were reported by Dunn and Fox Tree (2014) whose study made strides toward controlling for language mode effects. The study examined both English monolinguals and English-Spanish bilinguals who were divided into two groups before the experiment. Bilingual participants were randomly yet equally assigned to either the bilingual mode group or the monolingual mode group (consisting of monolinguals), and their bilingual language proficiency was first roughly assessed on an online survey during the online registration process (i.e., target questions about language ability were hidden in a variety of questions), and then was further assessed by a language dominance scale assessment and an individual interview after the completion of the experiment, minimizing the influence of confounding variables such as language dominance and language proficiency. Therefore, all participants had little reason to expect that their multilingual ability would be relevant for the study. The researchers also scheduled data collection sessions at times that minimized participants’ chance of encountering bilingual speakers or bilingual situations in the laboratory.

Dunn and Fox Tree’s (2014) study used a matched-pair design in the three experimental parts: in Part 1, all participants including English-speaking monolinguals and Spanish-English bilinguals were approached by a non-Latino experimenter and were asked to perform an English lexical decision with all instructions in English. In Part 2, all participants viewed a silent video clip about the Pink Panther and were asked to retell the story to the experimenter. In the monolingual mode group, since the need for Spanish was not mentioned, both the bilinguals and monolinguals should assume that their retellings be done in English, an assumption on which they acted corrected. However, in the bilingual mode group, bilinguals were approached by a Spanish-speaking experimenter. They were told in the instructions that Spanish retellings would enrich the database and were therefore asked to retell the story in Spanish. In Part 3, all participants performed another English lexical decision task. The results from the first lexical decision task (Part 1) showed that RTs did not differ between the two language mode groups. However, results from the second lexical decision task (Part 3) suggested that language mode significantly affected RTs such that bilinguals processed non-words slower in bilingual mode than in monolingual mode. Further analyses demonstrated similar RTs for English monolinguals and bilinguals in the monolingual mode. This finding not only supported the language mode hypothesis, but also appeared to diverge from Soares and Grosjean’s (1984) argument that bilinguals, regardless of which language mode they find themselves in, access words slower than monolinguals. It is likely that these differential results can be explained by the influence of several confounding factors in an experimental context in which participants were not fully in a monolingual mode.

In addition to the lexical decision task, evidence supporting the notion of language modes comes from picture-word interference tasks. Marian and Spivey (2003) found that in a monolingual mode, interference from the second language (L2) on the first language (L1) was not found, forming a contrast with the cross-linguistic effects while in a bilingual mode. However, interference from L1 to L2 was significant. Using the same paradigm, Canseco-Gonzalez et al. (2010) found that early Spanish-English bilinguals displayed inter-lingual competition that was significantly larger when they were tested in a bilingual mode (9.5% more fixations on the cohort than on the unrelated object) than in a monolingual mode (5%). These two studies suggest that not only language mode (environmental characteristics) but also participants’ characteristics such as language dominance and proficiency may modulate language activation. Importantly, the location on the language mode continuum will have a direct effect on language activation in terms of speech perception with inter-lingual competition becoming larger when the participants stay closer to the bilingual mode.

## Speech Production

Language mode also exerts considerable influence on bilinguals’ language production. Jared and Kroll (2001) simulated a monolingual mode in the first part of a picture-naming experiment and found little activation of the non-target language when participants expected to see only one language and were given stimuli and instructions in the same language. This consistently appeared to be the case except for when the non-target language was the participant’s dominant language. Nevertheless, when stimuli were given in both languages, as in Hermans et al. (2011), cross-language phonological co-activation appeared sensitive to the cognate status of the stimuli. These results supported the modulating effects of language mode: the higher the ratio of cognates to non-cognates, the higher the activation level of the non-target language.

In another study by Boukadi et al. (2015), Tunisian Arabic-French bilinguals named pictures in their L2 while ignoring auditory distractors. Bilingual participants were all native speakers of Tunisian Arabic who started learning French from primary school. Their L2 proficiency was assessed by means of self-ratings and a lexical decision task. In Experiment 1, the non-target language (Tunisian Arabic) was entirely absent in the experimental setting: all instructions were exclusively given in French and the students were not informed that the research was related to bilingualism until the end of the study. The participants were asked not to use their native language under any circumstances and to only communicate with the experimenter in French. The target stimuli were line-drawings of common objects and the auditory distractors were presented in French. Four French words were selected for each picture to serve as distractors based on the following conditions: phono-translation (the distractor was phonologically related to the picture name in L1); semantic (the distractor and target picture were semantically related); phonological (the distractor was phonologically related to the picture name in L1); and unrelated (the distractor had no relation to the picture name). No significant differences between the unrelated, phono-translation, or semantic condition were observed, which indicated that lexical selection proceeded in a language-specific way when the experimental setting was maintained in a monolingual mode. More importantly, the phono-translation effect remained insignificant even when L2 proficiency was taken into account. In Experiment 2, both languages appeared in the task in order to create a bilingual experimental setting, and bilinguals, who were selected from the same pool as Experiment 1, knew that the research had to do with a topic on bilingualism. They were allowed to speak in their L1 and were asked to name pictures in their L2 while ignoring an auditory distractor in their L1. Although the explicit instructions in Experiment 1 may have activated the irrelevant language, breaking a purely monolingual environment, Experiment 1 still created an environmental situation in which participants were closer to the monolingual end on the continuum compared with Experiment 2. In terms of the stimuli, Experiment 2 used the same pictures as in Experiment 1, but the auditory distracters were in Tunisian Arabic (the semantic distractors were the equivalent Tunisian Arabic translation of the French semantic distractors in Experiment 1). The results showed that RTs were significantly longer in both the phono-translation (965 ms) and semantic condition (934 ms) compared to the unrelated condition (918 ms). Taken together, the results of the two experiments suggest that language selection during bilingual speech production is a dynamic process modulated by language mode; the closer to the bilingual end of the continuum, the more activated the non-target language becomes. These findings also support the notion that the language mode of the experiment has modulating effects on the activation of bilinguals’ languages.

## Reading

Language mode also affects bilingual lexical access during word reading, as shown in a study carried out by Dijkstra et al. (2000). Dutch-English bilinguals with an average English learning time of 11.4 years participated in an English lexical decision task including English-Dutch homographs and cognates, as well as exclusively English control words. The total stimulus set was composed of homographs (no semantic similarity across languages), controls, English fillers, Dutch fillers, and non-words (orthographically permissible in English and not homophonic to Dutch words). The experiment included two parts, each consisting of 28 blocks of 8 stimuli including one homograph and one control item. In part 1, the remaining six item slots of each block were randomly filled with only English fillers or non-words, whereas in part 2, Dutch words were also included. The participants received the same instructions and communication with the experimenter (in English) for an English lexical decision task, but they were explicitly told that word forms that exist in both English and Dutch (homograph) required a “yes” response, while words only belonging to Dutch required a “no” response. After the experiment, all participants filled in a questionnaire to assess their L2 (English) proficiency. In this regard, part 1 of this experiment could be regarded as being close to a monolingual mode. The results showed that the RTs for homographs in part 2 were considerably slower (613 ms) than in part 1 (575 ms). This suggested that lexical selection took more time in the bilingual mode than in the monolingual mode and that participant moved closer to non-selective language activation. In addition, it should also be taken into account that the transition from part 1 to part 2 was rather abrupt, as RTs to interlingual homographs (from 581 to 663 ms) were considerably slower immediately after the transition. Consequently, encountering non-target language items during the experiment changes the language mode and exerts immediate and severe effects on bilingual lexical access during reading.

In Experiment 3 of a study by De Groot et al. (2000), the researchers mixed real words in the non-target language and non-words in the target language, forming a comparison with their Experiment 2 in which all the non-words were neither real words in the irrelevant language nor were they a mixture of the two languages. As a result, the participants responded to homographs faster in Experiment 2 (557 ms) than in Experiment 3 (619 ms). This provides support that the participants performed the task differently depending on the language mode simulated in the experiments: bilinguals processed words faster when the setting was more language-specific. In another study, Lemhöfer and Radach (2009) conducted a pure-German, a pure-English, and a mixed lexical decision task on the same set of non-words. Results showed that RTs varied according to the context of the task: in the monolingual context, participants made more mistakes and took longer to reject non-words that were more similar to the target language; in the bilingual context, RTs were significantly slower than RTs in the monolingual task with non-words that resembled the participants’ less-dominant language being harder to reject.

While some experiments have manipulated language mode by changing the composition of the stimuli, other studies have adjusted experimental settings of the task. Elston-Güttler et al. (2005) modified language mode by showing films with narration in different languages. They found that in an all-L2 sentence task with L2 pre-task priming (a film in the L2), RTs were significantly faster and decision thresholds were raised high enough to eliminate observable L1 influence on the L2. However, cross-linguistic interference was observed in the other experiment group who had L1 pre-task priming (a film in the L1). More recently, Khachatryan et al. (2016) manipulated the length of stimulus presentation in an L1 semantic priming task. Most of the subjects who saw stimuli presented for a shorter duration were aware of the presence of L2 manipulation, whereas none of the subjects in the other group were aware of this, placing the former group closer to bilingual mode and the latter group closer to monolingual mode. A significant facilitative effect of related word pairs in L2 was found when stimuli presentations were shorter but not when they were longer, indicating that the awareness of covert manipulation of L2 can influence the language mode and consequently what is measured in the laboratory. In short, these experiments suggest that both the selection of stimuli and the experimental contexts have the potential to modulate language activation in reading among bilinguals, and that the level of activation of the non-target language increases as the stimuli involve more words in the irrelevant language or as the setting moves closer to the bilingual context.

## Conflicting Findings

Although as shown above, several studies have reported on the role of language mode and its influence on language activation, there are contradictory findings. In our opinion, there are at least two possible explanations for these conflicting results. First, language activation may have been artificially induced by the experimental paradigms. Some experiments claim to have provided a “monolingual mode,” which in fact is an intermediate mode in disguise. Since language mode is quite sensitive to a wide range of factors, it takes lengthy efforts to create a purely monolingual environment, and therefore movement along the language mode continuum can be rather easy. For instance, according to previous studies (e.g., Hermans et al., 2011; Khachatryan et al., 2016), the subject’s awareness of the purpose of the study or a small proportion of cognate filler items suffice to activate the non-target language; hence making it arbitrary to assert the non-selectivity of language activation in all modes (see also Costa et al., 2000; Van Hell and Dijkstra, 2002; Duyck et al., 2007). Furthermore, the presence of speakers of the non-target language (e.g., bilingual experimenters or interlocutors with whom participants may come into contact), the language of all instructions, the discussion with or reports from other participants, and even a certain location may all artificially activate the non-target language to some extent, consequently moving bilinguals away from a purely monolingual mode.

Furthermore, research specifically testing participants’ and languages’ characteristics including language dominance, proficiency, and typology can explain some well-controlled yet conflicting experiments. Studies have found that language mode activation may vary when testing a dominant language vs. less-dominant language or when comparing balanced bilinguals to less-proficient bilinguals (e.g., Marian and Spivey, 2003; Lemhöfer and Dijkstra, 2004; Elston-Güttler et al., 2005; Lemhöfer and Radach, 2009; Dunn and Fox Tree, 2014). Moreover, variability in bilingual proficiency remains one of the main elements modulating non-target language activation and of the network responsible for language control (Green, 2011). According to Abutalebi and Green (2007), cross-language competition is greater among less-proficient bilinguals compared to highly proficient bilinguals which explains why in a pure monolingual mode (Colomé and Miozzo, 2010), the non-target language is invariably activated. In addition, multilinguals whose languages widely differ at lexical, grammatical, or phonological levels showed smaller interference effects as other multilinguals (van Heuven et al., 2011; Boukadi et al., 2015).

Green and Wei (2014) offer a similar account to speech planning and the cognitive processes involved in speech production, particularly in cases of code-switching. From a competitive account, Green and Wei (2014, p. 509) argue the importance of understanding “the interactional contexts of the bilingual speaker.” Bilinguals utilize processes that are most appropriate to certain situations and when they find themselves code-switching, these switches are “coordinated cooperatively and operate in a coupled or in an open-control mode. The former permits alternations and insertions whereas the latter is required for dense code-switching” (p. 499). For our purposes here, Green and Wei’s (2014) work implies that certain situations of multiple language use such as code-switching entail unique demands on control mechanisms and we could hypothesize the same for the unique demands needed as determined by many factors, including language mode.

To have a fully monolingual mode, it seems best to recruit both monolingual and bilingual participants so that the purpose of studying a topic related to bilingualism would not be revealed to the participants. Besides, during the experiment, the purpose of the study should always remain unknown (although it may be inadvertently disclosed after the critical experiments when asking about things like language proficiency or background). Alternatively, researchers can design several experiments to shift the participants’ focus away from the study’s purpose or they can invent a fictitious purpose as to prevent any activation of the irrelevant language. Ideally, participants should be recruited who have not academic knowledge of language selectivity, bilingualism, or language activation. During the experiment, all the experimental settings should be controlled carefully. For instance, the environment of the study (such as the language of the keyboard or computer system, posters on the wall, or any visible written words) should be strictly controlled. The experimenter should be highly proficient in the target language, preferably an L1 speaker and all experimental instructions should be given in that language as well. In addition, all materials (both visual and audio) for the study should be in the target language. The stimuli involved in lexical decision tasks should avoid any homographs or cognates and written words can be replaced with simple drawings in picture-naming tasks. In this regard, it is easier for researchers who work on two typologically different languages to simulate a more monolingual experimental setting, but the language competition between two different languages may be much weaker than that between two similar languages.

Taking language dominance and proficiency into consideration, it might be ideal to have a matched-pair design in order to make reliable comparisons with the bilingual mode. Consequently, the ideal location would be a place where two languages are equally used and the community attitude toward bilingualism should be positive. Ideally participants in monolingual and bilingual mode groups should be matched on their language proficiency in both languages, especially in L2. This can easily be done post-experiment by conducting a series of standardized tests on listening, writing, speaking, or reading abilities.

## Concluding Remarks

In line with Festman and Schwieter (2015) who argue that bilingual language control and activation should be studied using methods that include both mixed- and single-language experimental blocks, we would like to underscore here the importance of language mode as a confounding variable in studies looking at bilingual language activation and consequently, its implication for the cognitive benefits of bilingualism. Language mode is an important variable that modulates language activation. Simulating different points of the language mode continuum will elicit different results in studies of bilingual speech perception, production, and reading. It appears as though the more monolingual the language mode is, the more likely bilinguals will perform selective language processing. Consequently, language mode modulates language activation and alters the bilingual experience accordingly. However, language activation is also modulated by the interplay of several variables including task and participant characteristics making it challenging to create a pure monolingual mode in which selective language processing may occur. Language mode should be invariably considered as a potential and possible influence on multilingual experience. Given the importance and timeliness of this issue, future studies should specifically test the role that language mode plays in the bilingual experience and the modulating effects it may have on the cognitive benefits associated with bilingualism.

## Author Contributions

ZY and JS have contributed equally to the development of this paper.

## Conflict of Interest Statement

The authors declare that the research was conducted in the absence of any commercial or financial relationships that could be construed as a potential conflict of interest.

## Footnotes

1 By ‘degree of activation,’ we refer to the *magnitude* of language activation. While even today, it “remains to be determined what it means to say that languages can be activated to different degrees” (Dijkstra, 2005, p. 199), a recent study by Incera and McLennan (2018) found that differences in the timing of interference, but not in the magnitude of interference, led to differential effects within and between languages.

## References

[B1] Abutalebi J. Green D. (2007). Bilingual language production: the neurocognition of language representation and control. *J. Neurolinguistics* 20 242–275. 10.1016/j.jneuroling.2006.10.003

[B2] Barac R. Bialystok E. Castro D. Sanchez M. (2014). The cognitive development of young dual language learners: a critical review. *Early Child. Res. Q.* 29 699–714. 10.1016/j.ecresq.2014.02.003 25284958 PMC4180217

[B3] Bialystok E. (2015). Bilingualism and the development of executive function: the role of attention. *Child Dev. Perspect.* 9 117–121. 10.1111/cdep.12116 26019718 PMC4442091

[B4] Bialystok E. Craik F. Luk G. (2012). Bilingualism: consequences for mind and brain. *Trends Cogn. Sci.* 16 240–250. 10.1016/j.tics.2012.03.001 22464592 PMC3322418

[B5] Blom E. Boerma T. Bosma E. Cornips L. Everaert E. (2017). Cognitive advantages of bilingual children in different sociolinguistic contexts. *Front. Psychol.* 8:552. 10.3389/fpsyg.2017.00552 28484403 PMC5399246

[B6] Boukadi M. Davies R. Wilson M. (2015). Bilingual lexical selection as a dynamic process: evidence from Arabic-French bilinguals. *Can. J. Exp. Psychol.* 69 297–313. 10.1037/cep0000063 26372057

[B7] Canseco-Gonzalez E. Brehm L. Brick C. Brown-Schmidt S. Fischer K. Wagner K. (2010). Carpet or Cárcel: the effect of age of acquisition and language mode on bilingual lexical access. *Lang. Cogn. Process.* 25 669–705. 10.1080/01690960903474912

[B8] Colomé À (2001). Lexical activation in bilinguals’ speech production: language-specific or language-independent? *J. Mem. Lang.* 45 721–736. 10.1006/jmla.2001.2793

[B9] Colomé À Miozzo M. (2010). Which words are activated during bilingual word production? *J. Exp. Psychol. Learn. Mem. Cogn.* 36 96–109. 10.1037/a0017677 20053047

[B10] Costa A. Caramazza A. Sebastian-Galles N. (2000). The cognate facilitation effect: implications for models of lexical access. *J. Exp. Psychol. Learn. Mem. Cogn.* 26 1283–1296. 10.1037/0278-7393.26.5.1283 11009258

[B11] De Groot A. Delmaar P. Lupker S. (2000). The processing of interlexical homographs in translation recognition and lexical decision: support for non-selective access to bilingual memory. *Q. J. Exp. Psychol.* 53 397–428. 10.1080/713755891 10881612

[B12] Dijkstra T. (2005). “Bilingual visual word recognition and lexical access,” in *Handbook of Bilingualism: Psycholinguistic Approaches* eds Kroll J. de Groot A. (New York, NY: Oxford University Press) 179–201.

[B13] Dijkstra T. De Bruijn E. Schriefers H. Ten Brinke S. (2000). More on interlingual homograph recognition: language intermixing versus explicitness of instruction. *Biling. Lang. Cogn.* 3 69–78. 10.1017/S1366728900000146

[B14] Dunn A. Fox Tree J. (2014). More on language mode. *Int. J. Biling.* 18 605–613. 10.1177/1367006912454509

[B15] Duyck W. Van Assche E. Drieghe D. Hartsuiker R. (2007). Visual word recognition by bilinguals in a sentence context: evidence for nonselective lexical access. *J. Exp. Psychol. Learn. Mem. Cogn.* 33 663–679. 10.1037/0278-7393.33.4.663 17576146

[B16] Elston-Güttler K. Gunter T. Kotz S. (2005). Zooming into L2: global language context and adjustment affect processing of interlingual homographs in sentences. *Cogn. Brain Res.* 25 57–70. 10.1016/j.cogbrainres.2005.04.007 15905078

[B17] Festman J. Schwieter J. W. (2015). “Behavioural measures of language control: production and comprehension,” in *The Cambridge Handbook of Bilingual Processing* ed. Schwieter J. W. (Cambridge: Cambridge University Press) 527–547. 10.1017/CBO9781107447257.023

[B18] Green D. (1998). Mental control of the bilingual lexico-semantic system. *Biling. Lang. Cogn.* 1 67–81. 10.1017/S1366728998000133 29368141

[B19] Green D. (2011). Language control in different contexts: the behavioral ecology of bilingual speakers. *Front. Psychol.* 2:103. 10.3389/fpsyg.2011.00103 21779260 PMC3132677

[B20] Green D. Abutalebi J. (2013). Language control in bilinguals: the adaptive control hypothesis. *J. Cogn. Psychol.* 25 515–530. 10.1080/20445911.2013.796377 25077013 PMC4095950

[B21] Green D. W. Wei L. (2014). A control process model of code-switching. *Lang. Cogn. Neurosci.* 29 499–511. 10.1080/23273798.2014.882515

[B22] Grosjean E. (1998). Studying bilinguals; methodological and conceptual issues. *Biling. Lang. Cogn.* 1 131–149. 10.1017/S136672899800025X

[B23] Grosjean F. (2001). “The bilingual’s language modes,” in *One Mind, Two Languages: Bilingual Language Processing* ed. Nicol J. (Oxford: Blackwell) 1–22.

[B24] Hermans D. Ormel E. van Besselaar R. van Hell J. (2011). Lexical activation in bilinguals’ speech production is dynamic: how language ambiguous words can affect crosslanguage activation. *Lang. Cogn. Process.* 26 1687–1709. 10.1080/01690965.2010.530411

[B25] Hilchey M. Klein R. (2011). Are there bilingual advantages on non-linguistic interference tasks? Implications for the plasticity of executive control processes. *Psychon. Bull. Rev.* 18 625–658. 10.1075/lab.15041.fri 21674283

[B26] Incera S. McLennan C. (2018). The time course of within and between-language interference in bilinguals. *Int. J. Biling.* 22 88–99. 10.1177/1367006916644688

[B27] Jared D. Kroll J. (2001). Do bilinguals activate phonological representations in one or both of their languages when naming words? *J. Mem. Lang.* 44 2–31. 10.1006/jmla.2000.2747

[B28] Khachatryan E. Camarrone F. Fias W. Van Hulle M. (2016). ERP response unveils effect of second language manipulation on first language processing. *PLoS One* 11:e0167194. 10.1371/journal.pone.0167194 27893807 PMC5125703

[B29] Lemhöfer K. Dijkstra T. (2004). Recognizing cognates and interlingual homographs: effects of code similarity in language-specific and generalized lexical decision. *Mem. Cogn.* 32 533–550. 10.3758/BF03195845 15478748

[B30] Lemhöfer K. Radach R. (2009). Task context effects in bilingual nonword processing. *Exp. Psychol.* 56 41–47. 10.1027/1618-3169.56.1.41 19261577

[B31] Luk G. Bialystok E. (2013). Bilingualism is not a categorical variable: Interaction between language proficiency and usage. *J. Cogn. Psychol.* 25 605–621. 10.1080/20445911.2013.795574 24073327 PMC3780436

[B32] Marian V. Spivey M. (2003). Competing activation in bilingual language processing: within-and between-language competition. *Biling. Lang. Cogn.* 6 97–115. 10.1017/S1366728903001068

[B33] Paap K. Johnson H. Sawi O. (2015). Bilingual advantages in executive functioning either do not exist or are restricted to very specific and undetermined circumstances. *Cortex* 69 265–278. 10.1016/j.cortex.2015.04.014 26048659

[B34] Prior A. Gollan T. (2011). Good language switchers are good task-switchers: evidence from Spanish-English and Mandarin-English bilinguals. *J. Int. Neuropsychol. Soc.* 17 682–691. 10.1017/S1355617711000580 22882810

[B35] Schwieter J. W. Ferreira A. (2016). “Effects of cognitive control, lexical robustness, and frequency of codeswitching on language switching,” in *Cognitive Control and Consequences of Multilingualism* ed. Schwieter J. W. (Amsterdam: Benjamins) 193–216.

[B36] Soares C. Grosjean F. (1984). Bilinguals in a monolingual and a bilingual speech mode: the effect on lexical access. *Mem. Cogn.* 12 380–386. 10.3758/BF03198298 6503701

[B37] Spivey M. Marian V. (1999). Cross talk between native and second languages: partial activation of an irrelevant lexicon. *Psychol. Sci.* 10 281–284. 10.1111/1467-9280.00151

[B38] Van Hell J. Dijkstra T. (2002). Foreign language knowledge can influence native language performance in exclusively native contexts. *Psychon. Bull. Rev.* 9 780–789. 10.3758/BF03196335 12613683

[B39] van Heuven W. Conklin K. Coderre E. Guo T. Dijkstra T. (2011). The influence of cross-language similarity on within-and between-language Stroop effects in trilinguals. *Front. Psychol.* 2:374. 10.3389/fpsyg.2011.00374 22180749 PMC3238052

